# Kinetics of adaptive immune responses after administering mRNA-Based COVID-19 vaccination in individuals with and without prior SARS-CoV-2 infections

**DOI:** 10.1186/s12879-023-08728-5

**Published:** 2023-10-27

**Authors:** Sun-Woo Yoon, Kristin Widyasari, Jieun Jang, Seungjun Lee, Taejoon Kang, Sunjoo Kim

**Affiliations:** 1 Department of Biological Science and Biotechnology, Andong National University, Andong, 36729 Korea; 2 Gyeongsang Institute of Health Sciences, Gyeongsang National University, Jinju, 52727 Korea; 3 Gyeongnam Center for Infectious Disease Control and Prevention, Changwon, 51154 Korea; 4 Department of Laboratory Medicine, Gyeongsang National University Changwon Hospital, Changwon, 51472 Korea; 5 Bionanotechnology Research Center, Korea Research Institute of Bioscience and Biotechnology (KRIBB), Daejeon, 34141 Korea; 6 School of Pharmacy, Sungkyunkwan University, Suwon, 16419 Korea

**Keywords:** COVID-19, mRNA vaccine, Immune response, IFN-γ, Neutralizing antibody

## Abstract

**Objective:**

We aimed to compare the adaptive immune response in individuals with or without prior SARS-CoV-2 infections following the administration of mRNA-based COVID-19 vaccines.

**Methods:**

A total of 54 participants with ages ranging from 37 to 56 years old, consisting of 23 individuals without a history of SARS-CoV-2 infection (uninfected group) and 31 individuals with prior infection of SARS-CoV-2 (infected group) who have received two doses of mRNA SARS-CoV-2 vaccines were enrolled in this study. We measured the IFN-γ level upon administration of BNT162b2 (PF) or mRNA-1273 (MO) by QuantiFERON SARS-CoV-2. The production of neutralizing antibodies was evaluated by a surrogate virus neutralization assay, and the neutralizing capacity was assessed by a plaque reduction neutralization test (PRNT_50_). The immune response was compared between the two groups.

**Results:**

A significantly higher level of IFN-γ (p *<* 0.001) and neutralization antibodies (p *<* 0.001) were observed in the infected group than those in the uninfected group following the first administration of vaccines. The infected group demonstrated a significantly higher PRNT_50_ titer than the uninfected group against the Wuhan strain (p < 0.0001). Still, the two groups were not significantly different against Delta (p *=* 0.07) and Omicron (p *=* 0.14) variants. Following the second vaccine dose, T- and B-cell levels were not significantly increased in the infected group.

**Conclusion:**

A single dose of mRNA-based COVID-19 vaccines would boost immune responses in individuals who had previously contracted SARS-CoV-2.

**Supplementary Information:**

The online version contains supplementary material available at 10.1186/s12879-023-08728-5.

## Introduction

In December 2020, the first COVID-19 vaccine was administered [1]. This control measure was viewed as a solution to prevent the further spread of SARS-CoV-2 along with other prevention measures and to reduce mortality. BNT162b2 (PF) and mRNA-1273 (MO) are two messenger RNA (mRNA) vaccines that received approval from the U.S. Food and Drug Administration (FDA) and were among the first vaccines to receive WHO Emergency Use Listing (EUL), thus, have been widely used in many countries all around the world in the battle against COVID-19 [2–4]. PF and MO are administered as a 2-dose primary series [5, 6]. The mRNA vaccine is based on the principle that mRNA is an intermediate messenger delivering a transcript that encodes an antigen or immunogen. In contrast, the host’s immune response will recognize this antigen or immunogen [7, 8].

Since the first administration of mRNA-based COVID-19 vaccines, monitoring the safety and protection afforded by vaccines has become the most significant concern among the public. Studies on vaccine efficacy showed that PF and MO, as the administered COVID-19 vaccines, provide robust protection against COVID-19 [9–11]. A published study reported that the immune response in PF and MO vaccine recipients showed peak neutralizing-antibodies titer and T-cell response at 2–4 weeks after the second dose [9]. Given that mRNA-based vaccines provide promising protection against COVID-19 in healthy populations or populations with special conditions, administering mRNA vaccines to larger populations is crucial to reducing the incidence of severe illness from COVID-19.

As both SARS-CoV-2 infections and COVID-19 vaccination induce an adaptive immune response, the level and kinetics of the adaptive immune response following mRNA-based COVID-19 vaccination may vary in individuals with and without prior SARS-CoV-2 infections. Given that the adaptive immune response comprises cellular (T cell) and humoral (B cell) immune responses, we evaluated the kinetics of T- and B-cell responses to the mRNA-based COVID-19 vaccination in individuals with and without prior SARS-CoV-2 infections.

## Materials and methods

### Sample population

Fifty-four participants were enrolled in the study between June 2021 and February 2022. The participants were tested for COVID-19 infections by RT‒qPCR and distributed into two groups: healthy vaccinated (23 individuals) and infected vaccinated (31 individuals). All participants were Koreans with ages ranging from 37 to 56 years old. The healthy-vaccinated group, hereafter referred to as “uninfected,” is defined as a group of healthy individuals who have never been confirmed positive for SARS-CoV-2 by rapid antigen and RT-qPCR, never had any symptoms of SARS-CoV-2 infection, never had contact with SARS-CoV-2 infected individuals and received two doses of mRNA COVID-19 vaccines. The infected-vaccinated group, hereafter referred to as “infected,” is a group of participants who received two doses of mRNA COVID-19 vaccines after recovering from SARS-CoV-2 Wuhan strain infections for at least two months before being vaccinated. In this study, all participants received two doses of mRNA-based COVID-19 vaccines, either PF or MO. Participants with a history of specific allergies, pregnant women, or someone receiving immunosuppressants were excluded from the study. All participants agreed and submitted written consent for this clinical study.

### Sample collection

Blood samples were collected from the participants at three-time points (before the first dose and four weeks following the administration of the first and second doses of mRNA-based COVID-19 vaccines). A total of ten milliliters of blood was collected from each participant. For analysis of the T-cell response, 5 mL of whole blood was collected in a heparin tube and then transferred 1 mL for each of the QuantiFERON SARS-CoV-2 tubes (Nil, AG1, AG2, and mitogen tubes) (QIAGEN, Hilden, Germany). To analyze the B-cell response, 5 mL of whole blood was collected in a serum separation tube (SST) and centrifuged at 2,000 × g for 10 min. The obtained sera were aliquoted and stored at − 80 °C before the analysis.

### IFN-γ release assay (IGRA)

The IGRA assay was performed by QuantiFERON SARS-CoV-2, hereafter referred to as QFN SARS-CoV-2, which is based on in vitro activation of CD4 + and CD8 + T-cells in heparinized whole blood with a combination of specific SARS-CoV-2 antigens, followed by assessment of IFN-γ by enzyme-linked immunosorbent assay (ELISA) (QuantiFERON® ELISA) [12]. The epitope tubes comprise AG1 and AG2. The plasma sample from the mitogen tube served as the positive control, and the Nil tube was adjusted for the background (negative control). The assessment was conducted according to the manufacturer’s instructions. In brief, after the whole blood sample was distributed into QFN tubes, all tubes were incubated at 37 °C for 24 h and then centrifuged at 2,500 × g for 15 min to harvest the plasma. Furthermore, the obtained plasma was subjected to ELISA, which detects the IFN-γ (IU/mL) amount. The IFN-γ level of the Nil tube is subtracted from the IFN-γ level for the antigen tubes (AG1 and AG2) and the mitogen tube. A positive result was defined as a value of at least 0.20 IU/mL, more significant than the background from the Nil tube [13].

### Neutralizing antibody assay

The NAbs in the sera were detected using a cPass SARS CoV-2 surrogate virus neutralizing antibody test kit (GenScript, Piscataway, New Jersey, USA), hereafter referred to as the cPass sVNT. The cPass sVNT is an ELISA-based surrogate neutralization assay. This assay uses the primary interaction between the purified protein component of the SARS-CoV-2 receptor-binding domain (RBD) and human angiotensin-converting enzyme 2 (ACE2) in a competitive ELISA-based platform [14, 15].

To assess the NAbs, the cPass sVNT kit was used according to the manufacturer’s instructions. In brief, the sera of the participants were mixed with dilution buffer at a ratio of 1:10. The horseradish peroxide-conjugated recombinant SARS-CoV-2-RBD (HRP-RBD) solution was added and incubated at 37 °C for 30 min. The mixtures were subsequently incubated for 15 min at 37 °C in plates precoated with the ACE2 protein. After washing, the tetramethyl benzidine substrate solution (TMB) was added and incubated in the dark at 20 °C for 15 min. The stop solution was then added to halt the reaction, and the absorbance was read at 450 nm on an ELISA plate reader. The results were interpreted as positive according to the manufacturer’s recommendations when the inhibition value is ≥ the cutoff value (30%), indicating the presence of an anti-SARS-CoV-2 NAb.

### Plaque reduction neutralization test (PRNT)

Five samples from each group were arbitrarily selected for the plaque reduction neutralization test (PRNT). The wild-type SARS-CoV-2 (BetaCoV/Korea/KCDC03/2020), Delta variant (NCCP 43,390), and Omicron- B.1.1.529 (NCCP 43,408) were purchased from the National Biobank of Korea (Cheongju, Korea) and passaged three times in VERO-E6 cells (ATCC® CRL-1586™). For the PRNT assay, VERO-E6 cells were maintained in Dulbecco’s Modified Eagle’s Medium (Thermo Fisher Scientific, Waltham, Massachusetts, USA) supplemented with 2% fetal bovine serum (Thermo Fisher Scientific), penicillin (100 U/ml), and streptomycin (100 µg/ml; Thermo Fisher Scientific). The PRNT assay was performed using six-well culture plates (Thermo Fisher Scientific), with a twofold serial dilution of each serum sample incubated with 50 plaque-forming units (PFU) of each virus for 1 h at 37 °C. Each virus-serum mixture was added to monolayered VERO-E6 cells and incubated for 1 h at 37 °C before being overlaid on 1% agarose in cell culture media and incubated for 72 h. After incubation, the agarose was removed, and the cells were stained with 0.5% crystal violet to determine the plaque count. The PRNT_50_ titers of the serum sample were defined as the reciprocal of the highest serum dilution that resulted in a > 50% reduction in the number of virus plaques [16]. All experiments were conducted in the Biosafety Level 3 facility at the Korea Research Institute of Bioscience and Biotechnology by the standard operating protocols approved by the Institutional Biosafety Committee (IBC, approval number KRIBB-IBC-20,200,213).

### Statistical analysis

The differences in the IFN-γ concentration and the percentage of inhibition from the cPass sVNT assay in the uninfected and infected groups were assessed based on a repeated measures analysis of variance (RM-ANOVA). All statistical analyses for these assessments were two-tailed tests with a type I error of 5% and were performed using SAS software ver. 9.4 (SAS Institute Inc., Cary, NC, USA). The graph for the PNRT_50_ titer was built by GraphPad Prism v9.

## Results

### Cohort and study design

**Fig. 1:**
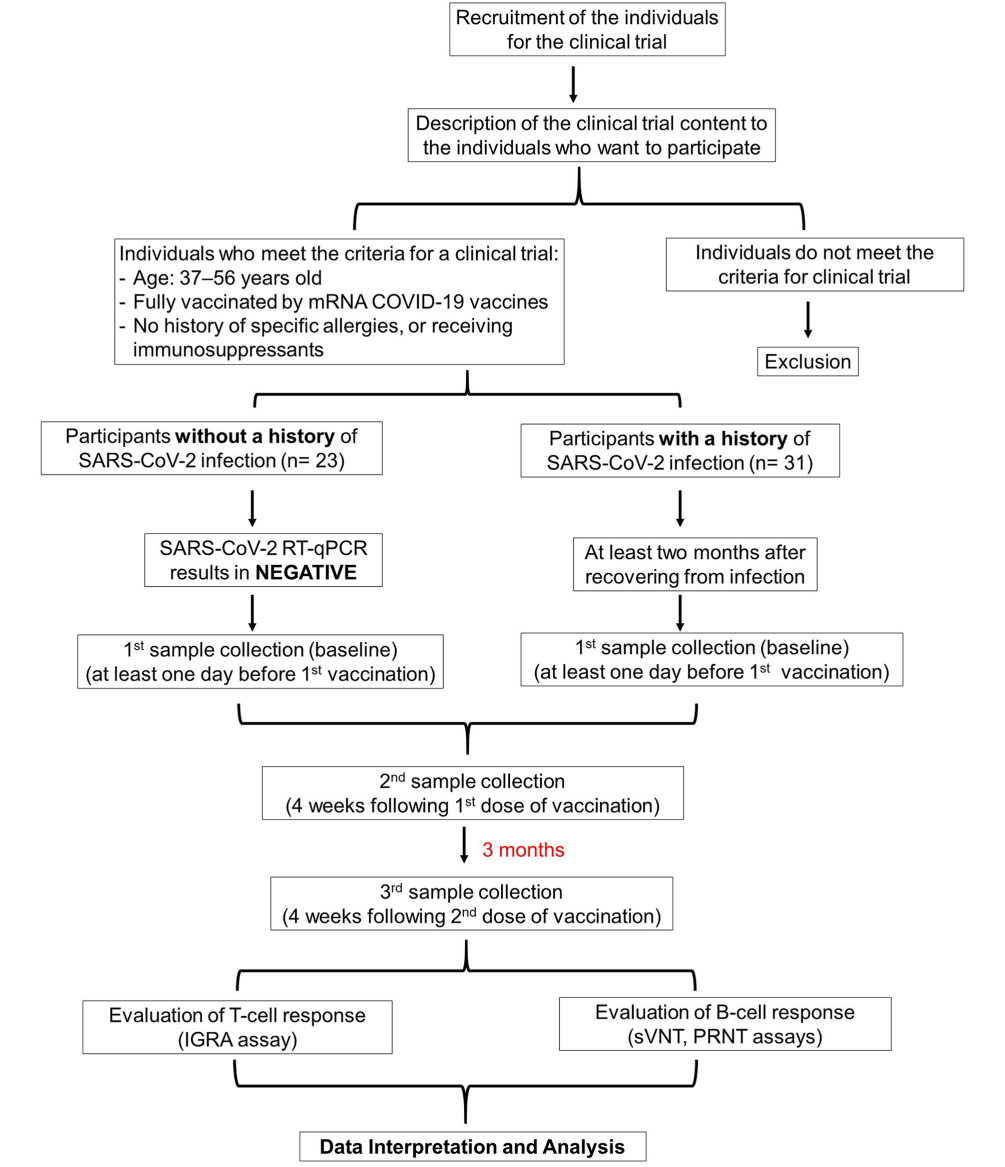
Diagram representing the study design

In total, 54 participants, 23 without and 31 with a history of COVID-19 at least two months prior (median 4, range 2–8 months) and who had received mRNA-based COVID-19 vaccines, were enrolled in this study (Fig. 1). The age range of the participants was 37–56 years old, with percentages of males 39.1% and 45.2% for uninfected and infected groups, respectively (Table 1). Among individuals in the uninfected group, 30.4% received PF and 69.6% received MO vaccination, while in the infected group, 40.6% received PF, and 50.0% received MO vaccination. Two participants (9.4%) in the infected group received mRNA-based COVID-19 vaccines; however, they could not recall which vaccine between PF or MO they had received (Table 1). Assessment of T- and B-cell responses to the specific SARS-CoV-2 antigen demonstrated a comparable level of immune responses between the PF and MO receivers in our previous study [17]. Moreover, considering the small sample size in our current study, classifying it into PF and MO groups would have been ineffective. Therefore, we combined PF and MO as an “mRNA-based COVID-19 vaccine”, and for further analysis, we focused our evaluation on uninfected and infected groups.

**Table 1:** Characteristics of study subjects

Characteristics	Uninfected population	Infected population
	N = 23	N = 31
**Sex**		
Male, N (%)	9 (39.1)	14 (45.2)
Female, N (%)	14 (60.9)	17 (54.8)
**Age (years), median (range)**	53 (37–54)	54 (47–56)
**Vaccine type**		
BNT162b2 (PZ), N (%)	7 (30.4)	13 (40.6)
mRNA-1273 (MO), N (%)	16 (69.6)	16 (50.0)
Not defined, N (%)	0 (0.0)	2 (9.4)

### Vaccination in individuals with or without prior SARS-CoV-2 infections boosted the production of IFN-γ

After the first dose of the mRNA-based COVID-19 vaccines was administered, the QFN SARS-CoV-2 assay for assessment of the T-cell response to the SARS-CoV-2 specific antigen revealed a high positive rate of up to 47.8% and 92.3% for the uninfected and infected groups, respectively. In the baseline, the positivity was 0% and 58.1% for uninfected and infected groups, respectively (Table 2). After the second dose of the mRNA-based COVID-19 vaccines was administered, the positivity rate of the tested sample increased even more, up to 95.7% and 95.2% for the uninfected and infected groups, respectively (Table 2).

**Table 2:** QuantiFeron (QFN) SARS-CoV-2 test positivity according to the vaccination schedule

QFN SARS-CoV-2	Uninfected population			Infected population		
	Baseline	1st dose	2nd dose	Baseline	1st dose	2nd dose
	N (%)	N (%)	N (%)	N (%)	N (%)	N (%)
**Negative**	23 (100.0)	12 (52.2)	1 (0.0)	13 (41.9)	1 (3.9)	0 (0.0)
**Positive**	0 (0.0)	11 (47.8)	22 (95.7)	18 (58.1)	24 (92.3)	20 (95.2)
**Intermediate** ^*^	0 (0.0)	0 (0.0)	0 (0.0)	0 (0.0)	1 (3.9)	1 (4.8)

^*^Intermediate refers to when the SARS-CoV-2 response and mitogen cannot be detected

**Fig. 2:**
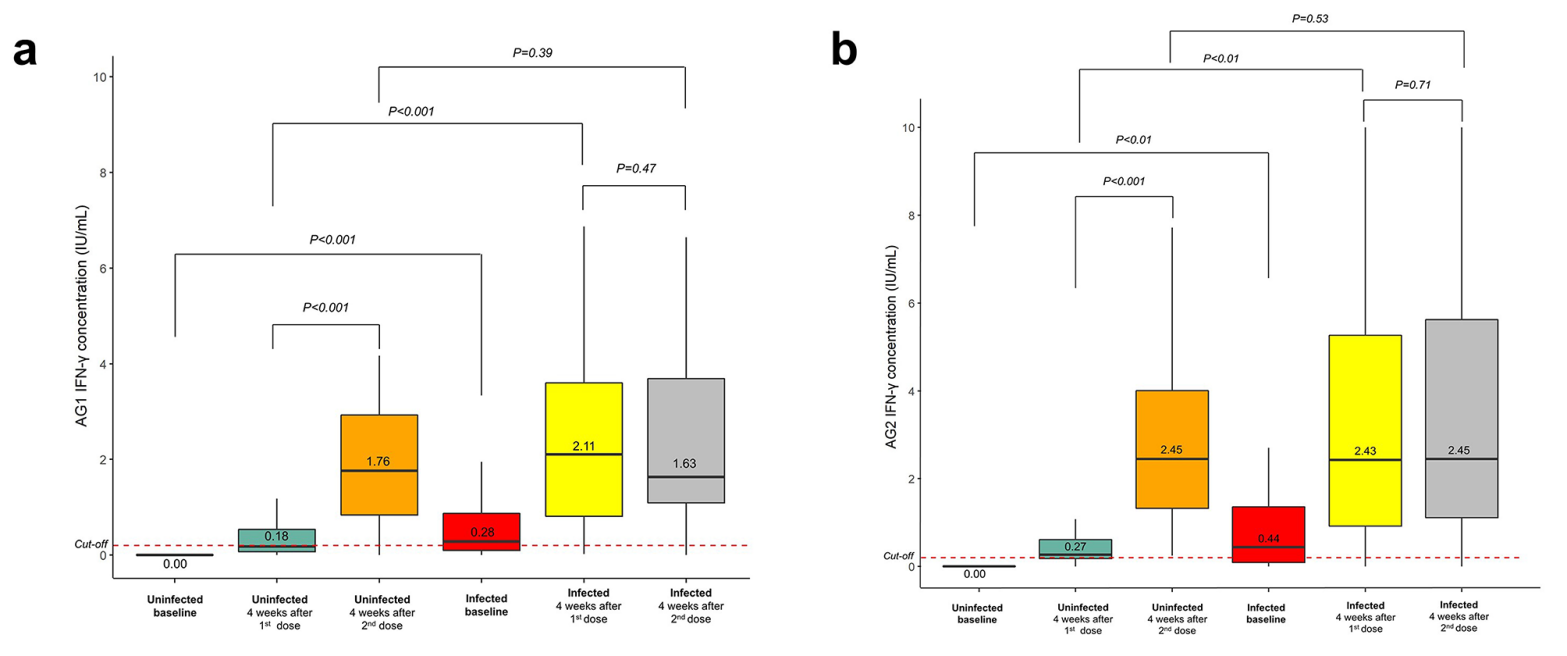
IFN-γ level after two doses of COVID-19 vaccines in individuals with or without prior SARS-CoV-2 infections. **(a)** The level of IFN-γ in response to the AG1 epitope. **(b)** The level of IFN-γ in response to the AG2 epitope. The dotted line indicates the cutoff value (0.2 IU/mL).

Administration of the first dose of mRNA-based COVID-19 vaccines increased IFN-γ in response to AG1 compared with the baseline up to a median 0.18 (interquartile range, IQR 0.07–0.57) IU/mL and 2.11 (IQR 0.80–3.63) IU/mL for uninfected and infected groups, respectively (Fig. 2a and Table S1). A similar pattern was also observed in the IFN-γ response to AG2 [median 0.27 (IQR 0.18–0.66) IU/mL and 2.43 (IQR 0.83–5.35) IU/mL] (Fig. 2b and Table S2). Following the administration of the second dose of mRNA-based COVID-19 vaccines, IFN-γ production in the uninfected group was significantly elevated compared with that after the first dose of vaccines, with a median of 1.76 (IQR 0.78–2.97) IU/mL, p < 0.001, whereas it declined slightly in the infected group [1.63 (IQR 1.09–3.69) IU/mL], p = 0.47, in response to AG1 stimulation (Fig. 2a and Table S1). Subsequently, in response to AG2 stimulation, the production of IFN-γ in the uninfected group was observed to be significantly higher than that after administration of the first dose of vaccine [median 2.45 (IQR 1.20–4.10) IU/mL, p < 0.001]. Meanwhile, in the infected group, the difference in IFN-γ production in response to AG2 stimulation after the second dose of vaccination was not significant [median 2.45 (IQR 1.11–5.62) IU/mL, p = 0.71] compared to that after the first dose (Fig. 2b and Table S2).

### Neutralizing antibodies are generated in uninfected and infected groups after administering mRNA-based COVID-19 vaccines

Evaluation of the neutralization antibody by the cPass sVNT assay demonstrated a high positivity rate after the mRNA-based COVID-19 vaccination. The positivity rate after the first dose of vaccines was up to 95.7% and up to 100% for the uninfected and infected groups, respectively. Subsequently, after administering the second dose, the positivity rate for both the uninfected and infected groups reached 100% (Table 3).

**Table 3:** GenScript cPass SARS-CoV-2 sVNT test positivity according to the vaccination schedule

cPass sVNT			Uninfected population			Infected population	
		Baseline	1st dose	2nd dose	Baseline	1st dose	2nd dose
		N (%)	N (%)	N (%)	N (%)	N (%)	N (%)
**Negative**		23 (100.0)	1 (4.3)	0 (0.0)	10 (32.2)	0 (0.0)	0 (0.0)
**Positive**		0 (0.0)	22 (95.7)	23 (100.0)	21 (67.7)	26 (100.0)	21 (100.0)

**Fig. 3:**
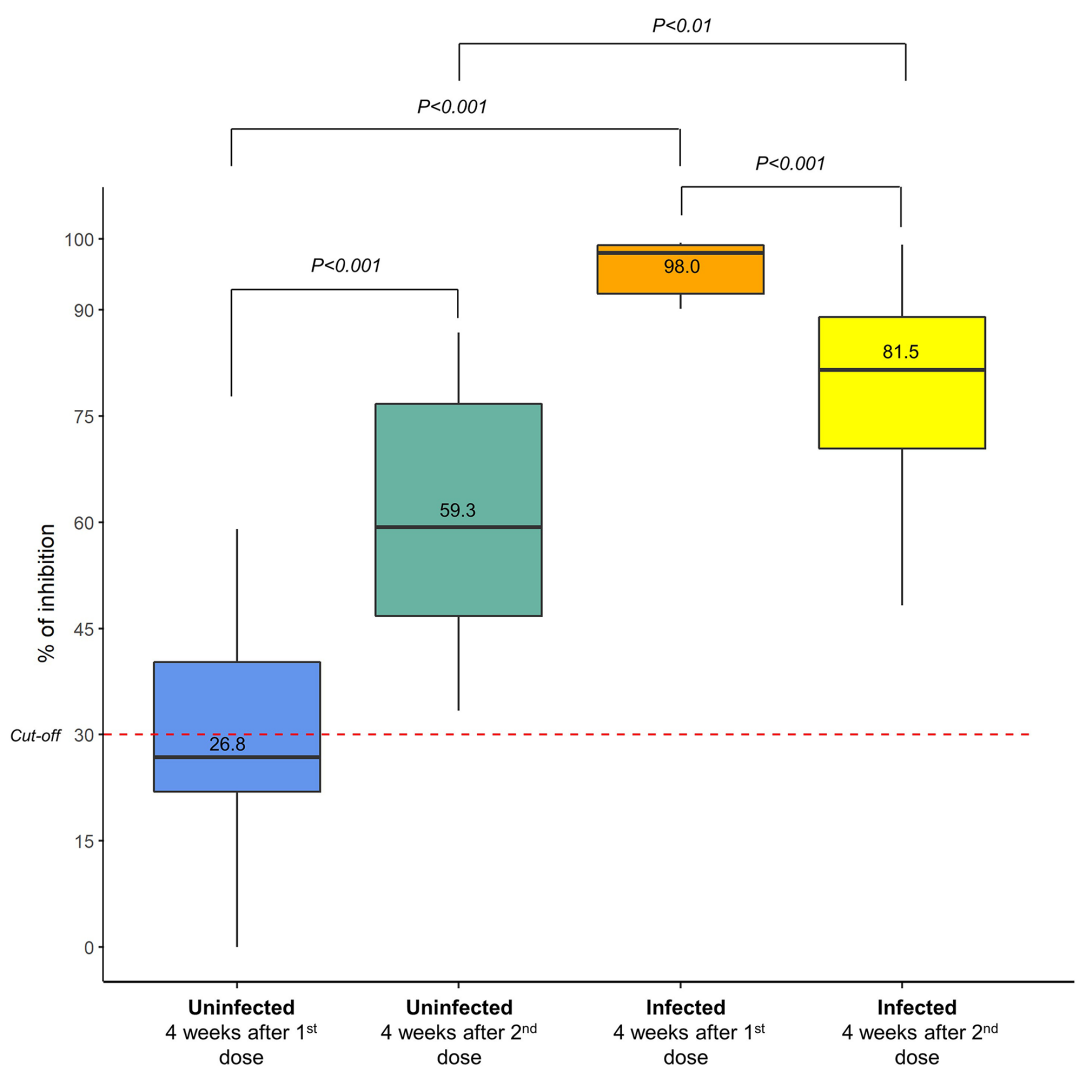
The percentage of inhibition after two doses of mRNA-based COVID-19 vaccines. The sera samples were diluted up to 1:20 before analysis. The dotted line indicates the cutoff value (30%)

Assessment of the neutralizing antibody by cPass sVNT showed that vaccination with mRNA-based COVID-19 vaccines triggered high inhibition in individuals with or without prior SARS-CoV-2 infections. In the preliminary experiment, we found that all tested sera yielded an exceedingly high percentage of inhibition (close to 100%); hence, comparing the percentage of inhibition among the groups using this result would have been ineffective. Using the sera that were diluted up to a ratio of 1:20, we found that the percentage of inhibition after administering the first dose of mRNA-based COVID-19 vaccines in the infected group was higher than that in the uninfected group: median 98.0 (IQR 91.5–99.1) % and 26.8 (IQR 20.0-40.5) % (p < 0.001), respectively (Fig. 3 and Table S3). A similar pattern was also observed after administering the second dose of mRNA-based COVID-19 vaccines, where the median percentage of inhibition was 81.5 (IQR 70.4–89.0) % and 59.3 (IQR 46.6–76.8) % (p < 0.01) for infected and uninfected groups, respectively (Fig. 3 and Table S3).

### Neutralizing responses vary against the original strain of SARS-CoV-2 and the variants of concern

We measured the neutralization efficiency of the neutralizing antibody from the serum samples against SARS-CoV-2 Wuhan strain, Delta, and Omicron- B.1.1.529 variants by PRNT_50_ assay. Five serum samples from each uninfected and infected group were selected and diluted from 1:20 to 1:640 before being subjected to the PRNT_50_ assay. Our assessment demonstrated that PRNT_50_ titers increased over the vaccine doses.

**Fig. 4:**
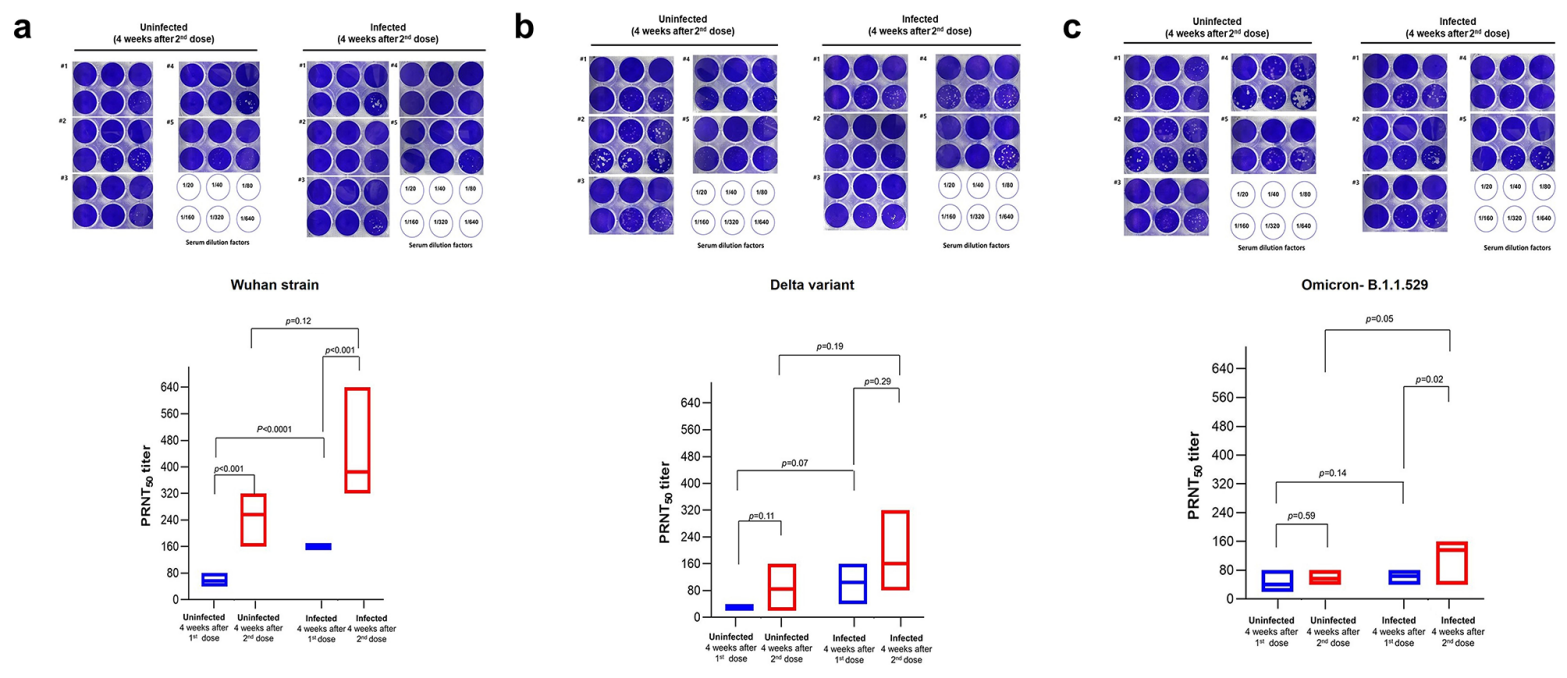
Neutralizing activity in individuals with or without prior SARS-CoV-2 infections. (**a**) representing neutralizing activity against the Wuhan strain, (**b**) against the Delta variant, and (**c**) against the Omicron variant. The upper panel represents the plaque reduction from each serum dilution. The lower panel demonstrates the PRNT_50_ titer

After administering the first dose of mRNA-based COVID-19 vaccine, the serum from the uninfected group was able to neutralize SARS-CoV-2 variants at a median dilution of 1:40, with the lowest dilution of 1:20 and the highest of 1:80. Meanwhile, the infected group appeared to have at least twofold higher PRNT_50_ titers, where the serum was able to neutralize SARS-CoV-2 variants at a median dilution of 1:80, with a range of dilution factors of 1:40 to 1:160. The serum’s ability from uninfected and infected groups to neutralize the virus was significantly higher after administering the second dose of vaccines than after administering the first dose. Nevertheless, the PRNT_50_ titer was observed to be higher in the infected group than in the uninfected group with the ability of serum to neutralize the SARS-CoV-2 variants at a median dilution factor of 1:160, with the lowest dilution factor of 1:40 and the highest of 1:640 (Fig. 4). In response to the variants of concern, the serum samples after administering the second dose of vaccines, either from the uninfected or infected group, demonstrated a high neutralizing ability to the Wuhan strains but appeared to be less effective against Delta or Omicron-B.1.1.529 variants [median dilution factors of 1:320, 1:160, and 1:160 for Wuhan strain, Delta, and Omicron-B.1.1.529 variants, respectively] (Fig. 4).

## Discussion

The adaptive immune response is crucial in controlling viral infections [18]. Monitoring the dynamics of the T- and B cells that comprise the adaptive immune response may help to determine the clinical course of viral infections and the effectiveness of vaccination. In this study, we demonstrated that the adaptive immune responses, both cellular (T cell) and humoral (B cell) immune responses, were elevated following the administration of COVID-19 mRNA vaccines in both uninfected and infected groups.

We observed that at four weeks following administration of the mRNA-based COVID-19 vaccines, the production of IFN-γ was significantly higher than before vaccination, suggesting the effectiveness of mRNA-based COVID-19 vaccines in wakening the adaptive immune response against SARS-CoV-2. We also observed that the IFN-γ concentration in the infected group was significantly higher after administering the first dose of vaccine than in the uninfected group. We assumed this condition occurs due to the cumulative effect of natural and acquired immunity, leading to higher T-cell responses in the infected group. In the uninfected group, the T-cell response is triggered by only a single antigen (spike protein). In contrast, in the infected group, the T-cell response may be triggered by the spike and non-spike proteins [19]. Thus, the epitope breadth of the T-cell response is broader and can substantially boost the T-cell response higher than in the uninfected group. This result is in concordance with the previously reported study, where the increase of the T-cell response in the vaccinated post-infection individuals was higher than in vaccinated naïve individuals [20]. Interestingly, unlike in uninfected individuals, administering the second dose of vaccines in infected individuals did not dramatically change the IFN-γ level. This suggests that individuals with prior infections may need only a single dose of mRNA-based COVID-19 vaccines to boost and maintain the immune response against SARS-CoV-2 [21, 22].

We also demonstrated that the administration of mRNA-based COVID-19 vaccines substantially triggered the production of neutralizing antibodies in both the uninfected and infected groups. The cPass sVNT assay showed that serum from the infected group yielded a significantly higher percentage of inhibition to SARS-CoV-2 than in the uninfected group following the first dose of the COVID-19 vaccine. After the second dose, however, we observed a decrease in the percentage of inhibition in the infected group. The factors associated with the decline in the percentage of inhibition after administering the second dose of initial COVID-19 vaccines are unknown. However, we hypothesize that this observation results from the primary immune response to natural infection. People with mild or severe COVID-19 infection are more likely to have developed SARS-CoV-2 specific antibodies, hence, one dose of vaccine may boost the immune response higher than in individuals who have only been vaccinated [23]. However, the second dose of vaccine possibly led to the functional exhaustion of spike-specific lymphocytes and did not further increase the B cell response [24]. Nevertheless, further study is required to elucidate the actual reason behind the decrease in B cell response after the second dose of the vaccine.

We also demonstrated neutralizing capacity of the serum samples against SARS-CoV-2 variants, in vitro. Our result showed that the neutralizing capacity of the serum samples against the Delta and Omicron variants was lower than against the Wuhan strain. This condition is likely due to the vaccines used in this study containing mRNA encoding the specific protein of the original Wuhan strains; hence, any changes in target antigen may affect the capacity of neutralizing antibodies elicited by the mRNA-based COVID-19 vaccines. Additionally, although we did not perform a genetic analysis for the virus from the infected group, the nationwide survey indicated that most were infected with the Wuhan strain during the infection period [25]. Hence, neutralizing antibodies triggered by the natural infection or vaccination were more effective against the Wuhan variant than the Delta and Omicron variants.

Several studies have described the ability of SARS-CoV-2 VOCs, particularly the Omicron variant, to escape NAbs elicited by initial COVID-19 vaccines [26–28]. The NAbs function by inhibiting binding between the receptor-binding domain (RBD) and angiotensin-converting enzyme 2 (ACE2). Any mutations in the RBD may reduce the neutralizing capacity of the NAbs. The Omicron variant contains at least 15 mutations in the RBD; hence, would efficiently evade the humoral immunity induced by prior infections or initial vaccination. This condition may trigger doubts about the effectiveness and importance of the initial mRNA-based COVID-19 vaccines. However, it is noteworthy that as demonstrated in our study and a published study [29], the initial mRNA-based COVID-19 vaccines elicited high T-cell responses that also contribute to giving protection against Omicron infection. The CD8 + T-cell plays an essential role in eliminating the already infected cells and controlling viral replication, particularly in the presence of insufficient antibodies. Meanwhile, the CD4 + T-cells provide signals that support the augmentation of antibodies [30, 31]. Unlike the B-cell response, the T-cell responses are persistent and last longer following vaccination and infection [32], and are not affected by the mutations [33]. For that reason, although the B-cell response elicited by the initial COVID-19 vaccines is likely less effective against the Omicron variant, the T-cell response elicited by the initial COVID-19 vaccines may continue to give protection against Omicron infection [29]. Therefore, our study contributed to the growing evidence of the importance of the initial mRNA-based COVID-19 vaccines in triggering cellular and humoral immunity changes in individuals with or without a history of SARS-CoV-2 infections.

Nevertheless, this study has some limitations. First, the in vitro T and B cell response assessment demonstrated an increased immune response following vaccination in the groups studied. However, a relatively small sample size for PRNT_50_ led to difficulties in determining the extent of serum neutralizing capacity against the variant of concern. Also, our inability to collect the blood samples over a longer duration after vaccination becomes another limitation of this study.

In conclusion, the changes in the adaptive immune response after vaccination observed in our study suggest the importance of administering a complete course (two doses) of mRNA vaccines in individuals without SARS-CoV-2 infection. Meanwhile, in individuals with a history of SARS-CoV-2 infection, one dose of vaccine may be adequate in triggering the adaptive immune response against the SARS-CoV-2 Wuhan variant to its peak.

### Electronic supplementary material

Below is the link to the electronic supplementary material.

Supplementary Material 1

## Data Availability

The datasets used and analyzed during the current study are available from the corresponding author upon reasonable request.
