# Supplementary material for: Kinetics of adaptive immune responses after administering mRNA-Based COVID-19 vaccination in individuals with and without prior SARS-CoV-2 infections
Source: BMC Infect Dis. 2023 Oct 27;23:732. doi: 10.1186/s12879-023-08728-5 (PMC10604405; doi:10.1186/s12879-023-08728-5)
Supplement: Supplementary file 1 — Supplementary Material 1 [file 12879_2023_8728_MOESM1_ESM.docx]

Supplementary Table 1. IFN-γ concentration (IU/mL) for QuantiFeron SARS-CoV-2 AG1 according to the vaccination schedule

| **Infection status** | **Vaccination** | **N** | **Median (interquartile range)** |
| --- | --- | --- | --- |
| **Uninfected population** | Baseline | 23 | 0.00 (0.00-0.01) |
|  | 1^st^ dose | 23 | 0.18 (0.07-0.57) |
|  | 2^nd^ dose | 23 | 1.76 (0.78-2.97) |
| **Infected population** | Baseline | 31 | 0.28 (0.09-1.03) |
|  | 1^st^ dose | 26 | 2.11 (0.80-3.63) |
|  | 2^nd^ dose | 21 | 1.63 (1.09-3.69) |

Supplementary Table 2. IFN-γ concentration (IU/mL) for QuantiFeron SARS-CoV-2 AG2 according to the vaccination schedule

| **Infection status** | **Vaccination** | **N** | **Median (interquartile range)** |
| --- | --- | --- | --- |
| **Uninfected population** | Baseline | 23 | 0.00 (0.00-0.01) |
|  | 1^st^ dose | 23 | 0.27 (0.18-0.66) |
|  | 2^nd^ dose | 23 | 2.45 (1.20-4.10) |
| **Infected population** | Baseline | 31 | 0.44 (0.08-1.53) |
|  | 1^st^ dose | 26 | 2.43 (0.83-5.35) |
|  | 2^nd^ dose | 21 | 2.45 (1.11-5.62) |

Supplementary Table 3. Neutralization antibody levels (%) measured by GenScript cPass SARS CoV-2 sVNT according to the vaccination schedule

| **Infection status** | **Vaccination** | **N** | **Median (interquartile range)** |
| --- | --- | --- | --- |
| **Uninfected population** | 1^st^ dose | 23 | 26.8 (20.0-40.5) |
|  | 2^nd^ dose | 23 | 59.3 (46.6-76.8) |
| **Infected population** | 1^st^ dose | 23 | 98.0 (91.5-99.1) |
|  | 2^nd^ dose | 21 | 81.5 (70.4-89.0) |
